# Visualizing Presynaptic Calcium Dynamics and Vesicle Fusion with a Single Genetically Encoded Reporter at Individual Synapses

**DOI:** 10.3389/fnsyn.2016.00021

**Published:** 2016-07-26

**Authors:** Rachel E. Jackson, Juan Burrone

**Affiliations:** Centre for Developmental Neurobiology, King’s College LondonLondon, UK

**Keywords:** presynaptic, calcium, neurotransmitter release, pHluorin, RGECO, vesicle

## Abstract

Synaptic transmission depends on the influx of calcium into the presynaptic compartment, which drives neurotransmitter release. Genetically encoded reporters are widely used tools to understand these processes, particularly pHluorin-based reporters that report vesicle exocytosis and endocytosis through pH dependent changes in fluorescence, and genetically encoded calcium indicators (GECIs) that exhibit changes in fluorescence upon binding to calcium. The recent expansion of the color palette of available indicators has made it possible to image multiple probes simultaneously within a cell. We have constructed a single molecule reporter capable of concurrent imaging of both presynaptic calcium influx and exocytosis, by fusion of sypHy, the vesicle associated protein synaptophysin containing a GFP-based pHluorin sensor, with the red-shifted GECI R-GECO1. Due to the fixed stoichiometry of the two probes, the ratio of the two responses can also be measured, providing an all optical correlate of the calcium dependence of release. Here, we have characterized stimulus-evoked sypHy-RGECO responses of hippocampal synapses *in vitro*, exploring the effects of different stimulus strengths and frequencies as well as variations in external calcium concentrations. By combining live sypHy-RGECO imaging with *post hoc* fixation and immunofluorescence, we have also investigated correlations between structural and functional properties of synapses.

## Introduction

Synaptic transmission depends on the influx of calcium into the presynaptic compartment, driving the release of vesicles containing neurotransmitter, which will bind to receptors on the postsynaptic cell. Electrophysiological studies have been fundamental in understanding the relationship between presynaptic calcium and neurotransmitter release (Dodge and Rahamimoff, 1967) but are limited in spatial resolution and cannot provide information about individual presynaptic terminals, unless employed at large synapses that can be accessed directly (Heidelberger and Matthews, 1992; Schneggenburger and Neher, 2000; Vyleta and Jonas, 2014). For studying individual synapses, optical strategies using either chemical indicators or genetically encoded reporters have therefore become the method of choice. Genetically encoded reporters offer the advantage that neural activity can be monitored in defined populations of cells, both *in vitro* and *in vivo*, and the probe can be localized to specific subcellular compartments (reviewed in Broussard et al., 2014).

The largest family of single color genetically encoded calcium indicators (GECIs) is the GCaMP family, based on a circularly permuted GFP linked to calmodulin (CaM) and it’s binding peptide M13 (Nakai et al., 2001). The original sensor has undergone multiple rounds of both random and structure-guided mutagenesis to improve stability, brightness, signal to noise ratio and kinetics (Tian et al., 2009; Akerboom et al., 2012; Chen et al., 2013) and several variants have been effectively targeted to the neuronal presynaptic terminal by fusion with synaptophysin (Dreosti et al., 2009; Li et al., 2011; Nikolaou et al., 2012). To enable multicolor imaging in single cells, red-shifted GECIs have been developed, including RCaMP (Akerboom et al., 2013) and R-GECO1 (Zhao Y. et al., 2011), for which a presynaptically targeted version (SyRGECO) has also been characterized (Walker et al., 2013). Much like the GCaMPs, red GECIs are constantly evolving, with new variants recently produced in both RCaMP and R-GECO families (Inoue et al., 2015; Dana et al., 2016).

The most widely used reporters of synaptic vesicle exocytosis and endocytosis consist of a synaptic vesicle protein fused to pHluorin, a pH-sensitive form of GFP (Miesenbock et al., 1998). Crucially in these constructs, pHluorin is localized to the acidic lumen of synaptic vesicles, so that its fluorescence is quenched at rest, but increases by up to 20-fold upon fusion with the plasma membrane and exposure to the more basic pH of the extracellular medium (Sankaranarayanan et al., 2000). Several variants exist including fusions to the intraluminal domains of VAMP2, known as synaptopHluorin, (Miesenbock et al., 1998), VGLUT1 (Voglmaier et al., 2006), and synaptophysin, known as sypHy (Granseth et al., 2006). Recently, efforts have been made to improve the normalization of presynaptic pHluorin responses, which are complicated by the presence of a fraction of the protein at the cell surface, particularly for synaptopHluorin and sypHy. At rest, surface stranded protein dominates the baseline fluorescence due to its increased brightness compared to quenched protein localized to synaptic vesicles and is highly variable between boutons, preventing the use of baseline fluorescence as a normalizing factor for exocytic responses. By fusing tdimer2 to the C-terminus of sypHy Rose et al. (2013) generated ratio-sypHy, in which the red fluorescence provides an invariant signal proportional to the expression of the probe rather than the surface fraction. The ratio of green to red fluorescence is fixed and allows normalization of sypHy responses to the red baseline, simplifying comparisons across cells with different expression levels and at different depths within a tissue. Finally, red-shifted reporters of vesicle fusion have also been generated, including VGLUT-2XmOr2 (Li et al., 2011), synaptobrevin 2-mOrange (Ramirez et al., 2012), and sypHTomato (Li and Tsien, 2012), again offering the possibility of combined imaging with other spectrally distinct reporters.

Despite the development of these tools, relatively few studies have been carried out in which both presynaptic calcium influx and vesicle exocytosis have been imaged in the same small, en-passant presynaptic terminals. Ermolyuk et al. (2012) used FM dyes to monitor vesicle exocytosis in conjunction with a calcium indicator introduced via a patch pipette, to examine the major parameters of presynaptic function including the size of the RRP, the probability of single vesicle fusion, calcium influx and the overall release probability of the synapse. Whilst this elegant study was highly informative, the techniques used are limited to relatively small numbers of cells and cannot be transferred to an *in vivo* situation. With genetically encoded reporters, visualization of both calcium influx and neurotransmitter release has been achieved by co-expression of a red-shifted reporter of vesicle exocytosis, either VGLUT-mOr2 or sypHTomato, with a green calcium indicator, such as members of the GCaMP family (Li et al., 2011; Li and Tsien, 2012). However, in this situation the two probes were expressed separately, resulting in possible differences in the level of expression of each reporter and in their subcellular location. Crucially, it also precludes the use of a ratiometric approach to measuring sypHy responses (Rose et al., 2013), complicating the direct comparison of responses across cells.

We have generated a new genetically encoded probe that brings together the measure of calcium and of exocytosis, in addition to a ratiometric measure of sypHy signals. To do this we combined sypHy, a well characterized pHluorin sensor of synaptic vesicle exocytosis (Granseth et al., 2006), with the red-shifted calcium indicator R-GECO1 (Zhao Y. et al., 2011) in a single molecule (sypHy-RGECO) to enable concurrent imaging of calcium influx and neurotransmitter release. The fixed 1:1 ratio of the two reporter molecules allows not only the normalization of sypHy signals to baseline or maximum RGECO fluorescence, but also the use of the ratio of the two responses as a measure of the calcium dependence of release at individual synapses. We systematically varied the number and frequency of action potentials, as well as the external calcium concentrations, and measured sypHy and RGECO signals in individual synapses. We found that while the amplitude of both responses changed, the distribution of the ratios remained constant. Interestingly, the calcium dependence of release was found to vary considerably between synapses, even within the same cell, suggesting the sensitivity of neurotransmitter release to calcium is synapse-specific. We also combined live sypHy-RGECO imaging with immunofluorescence for structural markers and observed that the amplitudes of calcium influx and exocytosis at individual synapses were positively correlated with the levels of the active zone protein RIM but were less well correlated with levels of the synaptic vesicle calcium sensor synaptotagmin-1.

## Materials and Methods

### Generation of syn::sypHy-RGECO Plasmid

SypHy was amplified by PCR using primers containing HindIII and NotI restriction sites from CMV::sypHy A4 (a gift from Leon Lagnado, Addgene plasmid # 24478). The resulting PCR fragment was subcloned in to a modified version of the pEGFP-N2 plasmid (Clontech Laboratories, USA), in which the CMV promoter had been replaced with the human synapsin I promoter. To amplify RGECO from SyRGECO (Walker et al., 2013), a forward primer containing an XmaI site followed by a 5 amino acid linker (GSGGT) was used in conjunction with a reverse primer containing a NotI site. When subcloned into these sites in the syn::sypHy-EGFP-N2 plasmid the EGFP was replaced to generate syn::sypHy-RGECO.

### Hippocampal Neuronal Culture and Transfection

Dissociated hippocampal neurons were prepared from Sprague–Dawley rats at embryonic day 18. Dissected hippocampi were treated with 5 mg/ml trypsin (Worthington, UK) for 5 min at 37 and triturated with fire-polished Pasteur pipettes. Neurons were plated on 18mm glass coverslips (Hecht Assistent, Germany) or 35 mm Grid500 μ-dishes (Ibidi, Germany) coated with poly-D-lysine (50 μg/ml) and laminin (20 μg/ml, both Sigma–Aldrich, UK). Cultures media were maintained in neurobasal media supplemented with 2% B27, 2% fetal bovine serum, 1% glutamax (all ThemoFisher Scientific, UK) and 1 % penicillin/streptomycin (Sigma), at 37°C in a humidified incubator with 5% CO_2_. After 3 days *in vitro* (DIV) the media was exchanged for serum-free media. At 7DIV neurons were transfected with sypHy-RGECO using Effectene (QIAGEN, UK). After transfection neurons were maintained in serum-free media without antibiotics. Only schedule 1 procedures performed by a competent individual were used in these studies, which are exempt under the Animals (Scientific Procedures) Act 1986.

### Imaging of sypHy-RGECO

Neurons were imaged at 17–21DIV in HEPES buffered saline (HBS; 139 mM NaCl, 2.5 mM KCl, 10 mM HEPES, 10 mM D-Glucose, 2 mM CaCl_2_, 1.3 mM MgCl_2_; pH 7.3, 290 mOsmol) with 2,3-dioxo-6-nitro-1,2,3,4-tetrahydrobenzo[*f*]quinoxaline-7-sulfonamide (NBQX), 0.025 mM amino-5-phosphonovaleric acid (APV) and 6-imino-3-(4-methoxyphenyl)-1(6*H*)-pyrida zinebutanoic acid hydrobromide (Gabazine) (all Tocris, UK). Coverslips were placed in a field stimulation chamber containing platinum electrodes (RC49-MFS, Warner Instruments, USA). For Ibidi dishes a stimulation insert (RC-37WS, Warner Instruments) was placed inside the dish. Neurons were imaged on an inverted Olympus IX71 microscope, equipped with a 60x/1.42 NA oil objective. Imaging was performed with a dual band-pass filter set optimized for EGFP and mCherry (Chroma, cat number 59022) and with LED excitation light sources of 470 and 585 nm (CoolLED, UK) for pHluorin and RGECO fluorophores, respectively. Time-lapse images were acquired at approximately 9.4 Hz using an Evolve 512 EMCCD camera (Photometrics, USA) controlled by Slidebook software (Intelligent Imaging Innovations, USA). Each frame consisted of a 20 ms exposure of the 470 and 585 nm LEDs sequentially, at 50 and 70% power, respectively, with 2 × 2 binning. Stimulation consisted of 1 ms 80 V pulses, which approximate single APs (Zhao C. et al., 2011), delivered by an SD9 Stimulator (Grass Technologies, USA), controlled by Slidebook. At the end of the imaging session 200 μM ionomycin was applied (Cayman Chemicals, USA) in either standard HBS, or HBS in which 50 mM of NaCl was replaced with 50 mM NH4Cl, to maximize sypHy-RGECO fluorescence. Neurons in Ibidi dishes were fixed in 4% PFA + 1% sucrose after imaging and were not treated with ionomycin. For calculations of surface fluorescence, synapses were imaged in HBS pH5.5 and HBS pH7.4 ±50 mM NH_4_Cl and the surface fraction calculated as (*G*_pH7.4_–*G*_pH5.5_)/(*G*_max_–*G*_pH5.5_). Synapses in which the surface fraction was calculated to be >100% were not included (<1% of synapses).

### Analysis of Live Images

Images were analyzed using custom written Matlab codes (Mathworks, USA). Regions of interest (ROIs, 6 × 6 pixels) were selected for each puncta of sypHy fluorescence. Mean background-subtracted fluorescence intensity values were calculated for each ROI in both sypHy (G) and RGECO (R) channels. Traces were smoothed by averaging over a sliding window of 4 frames. Baseline fluorescence (*G*_0_ and *R*_0_) was measured as the average of ten frames prior to the stimulus. ΔG and ΔR values were calculated by the change in signal intensity from the baseline, with the peak responses defined as the maximum ΔG and ΔR within 40 frames of the stimulus. Puncta in which the ΔR response to the first 10 AP 20 Hz stimulus was greater than three times the standard deviation of the baseline were considered responding synapses and were analyzed in further images or stimulation conditions. ΔG and ΔR values for each synapse were averaged across trials and any synapse in which the mean peak response did not reach 3 SDs of the mean baseline in either channel were also discarded. Before pooling, mean ΔG and ΔR responses were normalized to either *R*_0_ or *R*_max_, defined as the average of 10 images in 200 μM ionomycin.

### Immunofluorescence

After fixation cells were permeabilized with 0.2% Triton X-100 in PBS for 5 min, washed three times in PBS and placed in blocking solution (3% BSA in PBS + 0.05% NaN_3_) for 60 min at room temperature (RT), or for longer at 4°C. Cells were incubated in primary antibodies diluted in blocking solution for 60 min at RT then washed five times in PBS. Secondary antibodies in blocking solution were applied for 60 min at RT and cells were washed five times in PBS. Cells were mounted using Ibidi mounting medium.

Cells were stained with chicken anti-GFP (1:1000, Abcam, UK) to amplify the sypHy signal, and either rabbit anti-RIM1/2 (1:500, Synaptic Systems, Germany) or mouse anti Synaptogmin-1 (1:100, Synaptic Systems). Appropriate secondary antibodies tagged with Alexa 488 or 647 (ThermoFisher Scientific) were used at 1:1000.

### Fixed Cell Imaging and Analysis

The regions previously imaged live were located using the grid coordinates and imaged on an inverted Nikon Eclipse Ti spectral confocal microscope equipped with a 60x/1.40 NA oil objective using NIS Elements software. Excitation wavelengths were 405, 488, and 636 nm with bandpass emission filters set to 425–475 nm, 500–550 nm, and 662–737 nm. Microscope settings were kept the same for all images of the same antibodies. Z-stacks were generated from 0.15 μm optical sections, and maximum projections were produced in Image-J. To align the live and fixed images landmarks were selected in the GFP channel of both images, followed by affine transformation of the fixed image. ROIs from the live image were scaled and overlaid on the registered GFP image for manual confirmation of the position. The GFP image was then thresholded using the ‘Moments’ algorithm in ImageJ to produce a mask, which was applied to the other channels of the fixed image to avoid the inclusion of neighboring puncta in the ROI. Intensity values for each ROI were then calculated from the masked images.

### Statistical Analysis

Mean responses are displayed ±SEM unless otherwise stated, and *n* = the total number of synapses. As data are not normally distributed, non-parametric statistical tests have been used.

## Results

**FIGURE 1:**
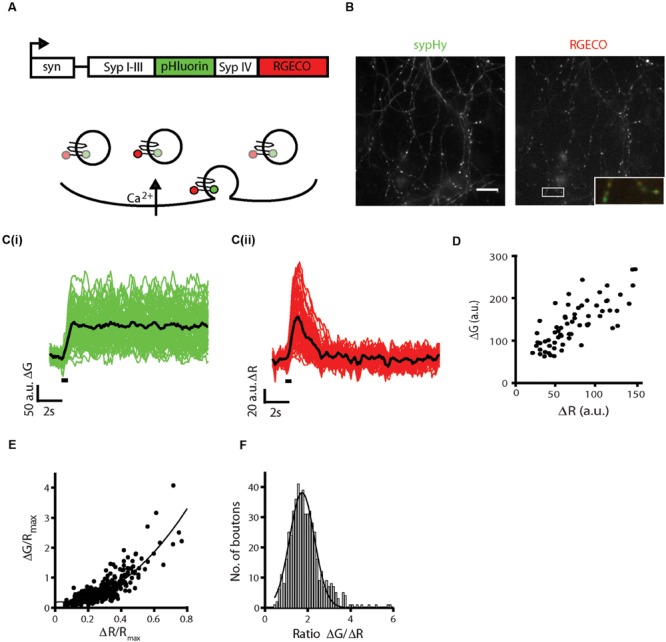
**Generation and characterization of sypHy-RGECO. (A)** Schematic of the sypHy-RGECO construct and its function. Presynaptic calcium influx is reported by an increase in RGECO fluorescence, situated on the cytoplasmic tail of synaptophysin, whilst exocytosis is reported by an increase in pHluorin fluorescence situated in the vesicle lumen. **(B)** Representative images of a dissociated hippocampal cell expressing sypHy-RGECO. Scale bar 20 μm. Inset shows merge of the two channels for the boxed region. **(C)** Changes in sypHy (**i**, ΔG) and RGECO (**ii**, ΔR) fluorescence in response to a field stimulus of 10 APs at 20 Hz. (*n* = 65 synapses from 1 coverslip, traces representing individual synapse responses shown in green/red, mean response of all synapses shown in black, stimulation period shown by black bar). **(D)** ΔG and ΔR responses are correlated (Spearman’s rank correlation *r*_s_ = 0.766, *p* < 0.001). **(E)** Cells were stimulated five times with a 10 AP 20 Hz stimulus. A mean response across trials was calculated and normalized to the maximum RGECO fluorescence in 200 μM ionomycin for each synapse. ΔG/*R*_max_ and ΔR/*R*_max_ responses from multiple cells show a non-linear correlation (*r*_s_ = 0.727, *p* < 0.0001, *n* = 526 synapses, from 9 coverslips and 5 independent cultures). The black line shows a fit to a power function of the form ax^b^+c, where *b* = 2.18. **(F)** Distribution of values for the ratio ΔG/ΔR, a measure of the calcium dependence of release (for the same synapses as in **E**).

To generate a single molecule reporter of both presynaptic calcium influx and synaptic vesicle exocytosis, the R-GECO1 fragment was amplified from SyRGECO (Walker et al., 2013) and subcloned downstream of sypHy (Granseth et al., 2006) with a short linker sequence. The whole sequence was placed under control of the human synapsin I promoter to drive expression in neurons. The resultant syn::sypHy-R-GECO1 plasmid will hereafter be referred to as sypHy-RGECO (**Figure 1A**). With this probe, presynaptic calcium influx is reported by an increase in red fluorescence and exocytosis of synaptic vesicles by an increase in green fluorescence (**Figure 1A**). In dissociated hippocampal neurons transfected with sypHy-RGECO, punctate fluorescence in presynaptic boutons was observed in both green and red channels (**Figure 1B**). A field stimulation of 10 action potentials (APs) at 20 Hz was applied and individual boutons responded with increases in fluorescence in both channels (**Figure 1C**), the amplitudes of which (ΔG and ΔR) were highly correlated (**Figure 1D**).

In order to pool responses from multiple cells or synapses, baseline fluorescence is often used to normalize reporter responses. However, for pHluorins this value can be dominated by protein localized to the plasma membrane, which is approximately 20-fold brighter than the quenched vesicular protein (Sankaranarayanan et al., 2000), and is highly variable between synapses. One method that has been used to overcome this problem involved fusion of the red fluorescent protein tdimer2 to the C-terminus of sypHy, generating a reporter termed ratio-sypHy (Rose et al., 2013). The fixed stoichiometry of the two fluorophores in this probe allowed normalization of the sypHy signals to the tdimer2 fluorescence. Using the same reasoning, RGECO can be used not only as a calcium indicator, but to normalize both ΔG and ΔR responses. As baseline RGECO fluorescence (*R*_0_) is relatively dim, we applied ionomycin in 2mM extracellular calcium to maximize the RGECO signal within the bouton (*R*_max_). Both values (*R*_0_ and *R*_max_) were highly correlated (Supplementary Figure S1A) and were subsequently used as a normalizing factor for sypHy and RGECO responses. After ionomycin treatment, sypHy fluorescence also gradually increased to a plateau presumably due to vesicle release caused by the large influx of calcium. The maximum fluorescence in green and red channels was also highly correlated, reflecting the fixed ratio of the two fluorophores at all synapses (Supplementary Figure S1B). A similar correlation was observed when ionomycin treatment was combined with application of HBS containing 50mM NH_4_Cl to alkalinize all compartments and maximize sypHy fluorescence (Supplementary Figure S1C). This contrasts with the less tight correlations observed between the baseline fluorescence values of the two channels, *G*_0_ and *R*_0_ (Supplementary Figure S1D), and between *G*_0_ and *G*_max_ (Supplementary Figure S1E), which are likely affected by heterogeneity in the baseline fluorescence of sypHy. To directly measure the surface fraction of sypHy-RGECO, we compared the difference in sypHy fluorescence in external solutions at pH5.5 and pH7.4 to the sypHy fluorescence maximized by application of 50mM NH_4_Cl. The surface fraction varied between synapses (Supplementary Figure S1F, mean = 30.8%, median = 24.8%) and was slightly higher than that reported for ratio-sypHy (median *f*_surf_ = 20%, Rose et al., 2013). This may be due to the fact that as basal RGECO fluorescence is low, it was necessary to identify transfected cells using basal sypHy fluorescence, potentially biasing toward brighter cells with higher surface fractions.

SypHy-RGECO responses to a 10 AP stimulus from multiple cells were normalized and pooled, and a positive non-linear correlation between ΔG/*R*_max_ and ΔR/*R*_max_ was observed (**Figure 1E**), as expected from the non-linear relationship between calcium and exocytosis (Dodge and Rahamimoff, 1967) and similar to that seen in other imaging studies (Zhao C. et al., 2011; Ariel et al., 2012; Ermolyuk et al., 2012). The spread of the responses suggested that individual synapses differed in the amount of neurotransmitter released for a given influx of calcium, which can be evaluated by the ratio ΔG/ΔR. We observed a range of ratio values, the majority of which fell within a normal distribution, although a small number of synapses displayed very high release in comparison to calcium influx, an order of magnitude higher than the lowest ratios (**Figure 1F**). Thus, there is heterogeneity in the calcium dependence of release between synapses.

When using ratiometric imaging, differential bleaching of the fluorophores could pose a potential problem. In boutons imaged without stimulation, sypHy fluorescence decayed by only 2% during the imaging period. This decay could be fit with a double exponential, with time constants of 1.64 s and 3172 s (Supplementary Figure S2A). Frames within the first time constant were therefore disregarded and frames from 1 s before the stimulus were used to calculate baseline fluorescence. The slow phase of bleaching, which accounted for 98.5% of the decay, had a negligible effect on either baseline fluorescence or maximum ΔG amplitude and was not corrected for, although this would need to be considered if imaging over a long period or using this probe to measure vesicular endocytosis rates. RGECO did not exhibit bleaching, instead showing an initial rapid increase in fluorescence that plateaued, the reasons for which will be discussed in more detail below (Supplementary Figure S2B). The rapid initial changes in fluorescence suggested that sequential images may be affected by differential bleaching. To test this, cells were stimulated with 10 AP stimuli five times, with a 1 min interval between images. The average ΔG and ΔR responses both decreased over the trials, with a slightly greater decrease in the calcium response (Supplementary Figure S2C). The reasons for these decreases are not clear, and may be different for the two responses. RGECO did not exhibit bleaching across trials, whilst the baseline fluorescence of sypHy decreased by approximately 20% (Supplementary Figure S2C). It has previously been suggested that GECIs targeted to the presynaptic compartment can exhibit rundown in responses (Walker et al., 2013), whereas the responses of synaptic pHluorins are similar across repeated trials (Burrone et al., 2006). However, the ratio of the two responses remained relatively stable across trials, increasing by only 9% between the first and fifth repeat (Supplementary Figure S2C). To avoid any inherent bias due to the effect of repeated trials we interspersed different stimulation conditions, such as number or frequency of AP stimuli.

It has previously been reported that R-GECO1 displays photoactivation when illuminated with 488 nm light (Akerboom et al., 2013), which may explain the increase in red fluorescence observed in unstimulated boutons (Supplementary Figure S2B). To examine if this phenomenon affected sypHy-RGECO calcium responses, single channel images, illuminated with 585 nm light only, were compared to our usual imaging conditions, alternating 470 and 585 nm light. ΔR responses were larger in images taken with 585 nm light only (Supplementary Figure S2C, upper trace), although when scaled for comparison, the kinetics of the response were the same in both imaging conditions (Supplementary Figure S2C, lower trace). The response amplitudes and baseline fluorescence in single channel and two channel images were highly correlated and, importantly, scaled linearly (Supplementary Figures S2D,E). These results suggested that in our imaging conditions, fast alterations between blue and green excitation light reduces the size of the RGECO responses in a multiplicative manner, without the introduction of non-linear artifacts.

**FIGURE 2:**
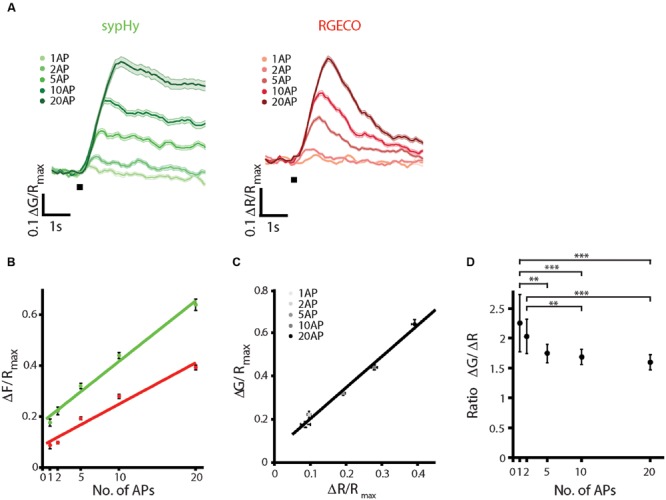
**SypHy-RGECO responses to different stimulus strengths.** A range of AP stimuli were delivered by field stimulation at 20 Hz. Each stimulus was repeated twice, varying the order in which different numbers of APs were given, and a mean of the two responses was taken. **(A)** Mean sypHy and RGECO responses to different numbers of APs, the black bar indicates the start of stimulation. **(B)** Peak ΔG/*R*_max_ (green) and ΔR/*R*_max_ responses (red) increase linearly over the range of AP stimuli tested (colored lines indicate linear regression fit: sypHy, green, *r*^2^ = 0.986, RGECO, red, *r*^2^ = 0.961). **(C)** Correlation between peak ΔG/*R*_max_ and ΔR/*R*_max_ responses over the range of APs tested (black line indicates linear fit, *r*^2^ = 0.989). **(D)** Ratio ΔG/ΔR for each of the stimulus strengths tested. Only the ratios for 1 and 2 APs show a significant difference from the other stimuli (one way ANOVA, ^∗∗^*p* < 0.01, ^∗∗∗^*p* < 0.001). All plots show mean ±SEM, *n* = 171 synapses from 4 coverslips and two independent cultures.

To characterize the sypHy-RGECO response to different stimulus strengths, stimuli ranging from 1 AP to 20 AP at 20 Hz were delivered by field stimulation. Both responses increased linearly over the range of stimulus strengths tested (**Figures 2A,B**), as has been previously reported for SyRGECO (Walker et al., 2013). Peak ΔG and ΔR responses to different numbers of action potentials also showed a strong linear correlation (**Figure 2C**). The mean ratio between vesicle exocytosis and calcium influx did not differ between 5, 10, and 20 AP stimuli (**Figure 2D**). The mean ratio was significantly increased at lower stimulus strengths, although many synapses that responded at higher stimulus strengths fell below the detection threshold in the 1 AP and 2 AP stimuli, potentially biasing toward synapses with higher release probabilities in these groups. At higher numbers of APs, the longer stimulation times required would begin to overlap with the endocytosis and reacidification of synaptic vesicles, making the amplitude of sypHy responses harder to interpret. However, to check for potential saturation of the probe we also delivered 40 and 100 AP stimuli at 20 Hz. The calcium response indicated by RGECO was saturated at 40 APs and displayed no further increase in amplitude to a 100 AP stimulus, whilst sypHy responses continued to increase with longer stimulation trains (Supplementary Figure S3).

**FIGURE 3:**
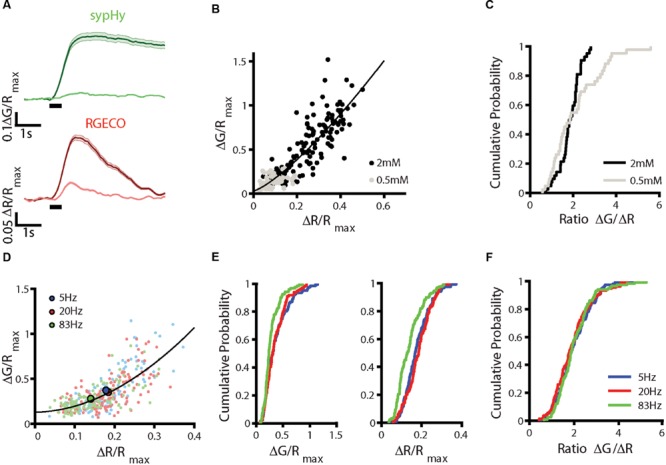
**Effects of altering stimulation conditions. (A–C)** Dissociated hippocampal neurons in external solution (HBS) containing 0.5 mM calcium were stimulated with a train of 10 APs at 20 Hz repeated five times 1 min apart. The solution was replaced with HBS containing 2 mM calcium and the stimulus set repeated. Responses across trials were averaged for each synapse. **(A)** Fluorescence changes in sypHy (upper traces) and RGECO (lower traces) in 0.5 and 2 mM. Traces show mean ± SEM for *n* = 131 synapses from 3 coverslips from 3 independent cultures. **(B)** Correlation between peak ΔG/R_max_ and ΔR/R_max_ responses for these synapses (Spearman’s rank correlation *r*_s_ = 0.873, *p* < 0.001, the black line indicates a fit to a power function of the form ax^b^+c, where *b* = 1.30.) **(C)** Distribution of ΔG/ΔR ratios for synapses which responded above threshold at both calcium concentrations, ratios shown are averaged from the last two 0.5 mM trials (gray) and the first two 2 mM trials (black) (*n* = 47 synapses). The distribution is not significantly different between concentrations (Kolmogorov–Smirnov test, *p* = 0.093). **(D–F)** Hippocampal neurons were stimulated with 10 APs at 5, 20, and 83 Hz. Each set of stimuli was repeated three times, varying the order in which the frequencies were delivered and the mean of the trials was calculated. **(D)** Correlation between peak ΔG/*R*_max_ and ΔR/*R*_max_ values at 5 Hz (blue), 20 Hz (red), and 83 Hz (green). Mean responses to each frequency are shown in the larger, brighter points (*n* = 134 synapses from 3 coverslips and three independent cultures, the black line indicates a fit to a power function of the form ax^b^+c, where *b* = 1.91). **(E)** Distribution of ΔG/*R*_max_ (left) and ΔR/*R*_max_ (right) responses for these synapses show a decrease in the responses at 83 Hz compared to 20 Hz (Kolmogorov–Smirnov test, ΔG/*R*_max_ *p* = 0.0013, ΔR/*R*_max_ *p* < 0.001) but no change in the response at 5 Hz (Kolmogorov–Smirnov test ΔG/*R*_max_ *p* = 0.537, ΔR/*R*_max_ *p* = 0.240). **(F)** The distribution of ΔG/ΔR ratios does not change between stimulus frequencies (Kolmogorov–Smirnov test 5 Hz *p* = 0.703, 83 Hz *p* = 0.282).

We next tested the effect of changing the external calcium concentration, recording sypHy-RGECO responses in both 0.5 mM and 2 mM calcium for the same set of synapses. As expected, both the calcium influx and level of vesicle release were significantly reduced in 0.5 mM calcium (**Figure 3A**), although the positive correlation between the two responses was maintained (**Figure 3B**). For synapses which responded above threshold in both conditions, the distribution of ΔG/ΔR ratios was not significantly different between the two concentrations (**Figure 3C**), although the ratios measured for 0.5 mM calcium showed a skewed distribution toward higher values. This discrepancy is likely due to the saturation of the vesicular sensor of exocytosis for higher calcium concentrations, as reported previously (Ermolyuk et al., 2012).

Calcium influx at the presynaptic terminal is predominantly mediated by Ca_V_2.1 and 2.2 channels, with some contribution from Ca_V_2.3 (Takahashi and Momiyama, 1993; Wheeler et al., 1994; Gasparini et al., 2001). The contribution of different channel subtypes to the presynaptic calcium signal varies not only between synapses, but also with stimulation frequency (Ricoy and Frerking, 2014). We stimulated neurons expressing sypHy-RGECO with 10 APs at 5, 20, and 83 Hz, to examine whether stimulation frequency affected the relationship between ΔG and ΔR responses. Pooled ΔG and ΔR responses across all frequencies showed the expected non-linear relationship (**Figure 3D**). Both calcium influx and vesicle exocytosis were significantly reduced at 83 Hz stimulation, which could be due to the failure of the cell to fire action potentials at high frequency, or to the inactivation of calcium channels (**Figures 3D,E**). However, the distributions of ΔG/ΔR ratios were indistinguishable between stimulation frequencies (**Figure 3F**), suggesting frequency did not alter the relationship between calcium influx and vesicle release.

**FIGURE 4:**
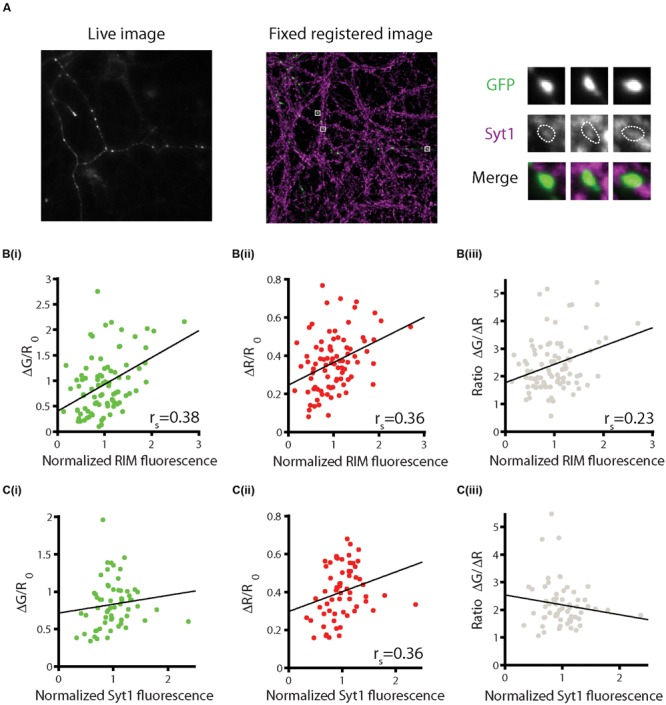
**Combining functional imaging with sypHy-RGECO and immunofluorescence.** Neurons were stimulated five times with 10 APs at 20 Hz and the responses averaged across trials. Cells were then fixed and stained for presynaptic markers and imaged using confocal microscopy. Live and fixed images were registered and fluorescence intensities for individual synapses were measured. **(A)** Example of a live image of the green channel of sypHy-RGECO (left) and fixed image (middle) of GFP and synaptotagmin-1 (Syt1) for the same cell. Right, magnified view of individual synapses. **(B)** Positive correlations between levels of RIM and sypHy responses (**i**, green, *p* < 0.001), RGECO responses (**ii**, red, *p* < 0.001), and the ΔG/ΔR ratio (**iii**, gray, *p* = 0.039), (*n* = 85 synapses from 3 dishes from independent cultures). **(C)** Levels of Syt1 are not significantly correlated with sypHy responses (**i**, *p* = 0.059), or the ΔG/ΔR ratio (**iii**, *p* = 0.323), but are correlated with the calcium response (**ii**, *p* = 0.005) (*n* = 62 synapses from three dishes from independent cultures).

Synaptic boutons are heterogeneous in both structural and functional parameters. Studies of the relationship between the two show that bouton volume is a poor predictor of functional properties such as presynaptic calcium influx and the synaptic probability of release (Ermolyuk et al., 2012; Holderith et al., 2012), whereas active zone size strongly correlates with function (Holderith et al., 2012). Additionally, levels of the active zone proteins RIM1/2 are highly correlated with active zone size (Holderith et al., 2012). To examine the relationship between sypHy-RGECO responses and active zone size, neurons were first imaged live, whilst stimulating with 10 APs at 20 Hz, and subsequently fixed and stained for RIM1/2. Live and fixed images were aligned to allow the comparison of functional and structural data from individual identified synapses (**Figure 4A** and Supplementary Figure S4). We observed significant positive correlations between RIM levels and both sypHy and RGECO responses (**Figure 4Bi,ii**). Whilst significant, these correlations are weaker than those found in ultrastructural studies, most likely due to limitations in the alignment process and the lower resolution of light microscopy. Interestingly, RIM levels also correlated significantly, albeit weakly, with the ratio ΔG/ΔR (**Figure 4Biii**), possibly reflecting the role of RIM, along with RIM binding protein (RBP), in localizing calcium channels to the active zone (Kaeser et al., 2011), vesicle docking (Han et al., 2011) and coupling of calcium channels to release (Kaeser et al., 2012).

Similar correlations were also carried out with staining for the putative calcium sensor for synchronous neurotransmitter release, synaptotagmin-1 (Syt1) (Geppert et al., 1994; Fernandez-Chacon et al., 2001). We found that the levels of Syt1 were positively correlated with the magnitude of the RGECO response, and showed a trend toward a positive correlation with neurotransmitter release that did not reach significance (**Figures 4Ci,ii**). However, the ratio of release to calcium influx at each synapse was not related to the level of Syt1 (**Figure 4Ciii**). As the neurons were stimulated with a train of APs at high frequency (20 Hz), both synchronous and asynchronous forms of release are expected to operate (Hagler and Goda, 2001), and thus the levels of other calcium sensors may be more important in determining the total release that occurs in response to this type of stimulus. Furthermore, approximately 20% of total Syt1 is found on the surface of the bouton, not on vesicles (Fernandez-Alfonso et al., 2006), thus the level of Syt1 present at the bouton may not accurately reflect the size of the vesicle pool. Together, our data is in broad agreement with the general principle that synapses that have larger active zones also show higher levels of calcium influx and neurotransmitter release. Although the overall levels of Syt1 at the synapse also broadly correlated with these functional measures, they were a worse predictor of synapse function than RIM.

## Discussion

We have generated a single molecule reporter, sypHy-RGECO, for concurrent imaging of presynaptic calcium influx and synaptic vesicle recycling. This hybrid molecule consisted of a red calcium indicator, R-GECO1 (Zhao Y. et al., 2011), fused to the C-terminus of sypHy, a reporter of vesicle exocytosis and endocytosis (Granseth et al., 2006). Combining the two reporters in one molecule offers several advantages. First, it provides two independent readouts of presynaptic function, without the need for co-expression of multiple probes. Second, as the probes are not separated spatially and a fixed ratio of the two fluorophores is present at all synapses, it provides a way of normalizing sypHy signals across synapses and cells, in a manner similar to that employed for ratio-sypHy (Rose et al., 2013). This is particularly important for sypHy signals, where resting levels of fluorescence vary quite dramatically from bouton to bouton and do not correlate directly with the amount probe at the synapse, but rather to its surface levels. Finally, the fixed stoichiometry also allows a direct comparison of the two fluorescence responses, thereby providing a direct measure of the calcium dependence of release within single presynaptic boutons.

We observed a strong non-linear correlation between calcium influx and vesicle exocytosis across synapses that was maintained in response to different stimulation conditions, in agreement with many previous studies (Ariel et al., 2012; Ermolyuk et al., 2012). Using the ratio of the sypHy response to the RGECO response (ΔG/ΔR), we were also able to measure the calcium-dependence of release at individual presynaptic boutons, a feature that has been difficult to study in these small CNS synapses. Interestingly, we observed heterogeneity in this value, suggesting that calcium triggers vesicle fusion with different efficiencies at different synapses. It is not yet clear what underlies this variation, as the distribution of ΔG/ΔR ratios did not change significantly under different stimulation conditions, such as stimulation frequency and extracellular calcium, despite changes in the amplitude of both calcium and exocytosis signals. A possible source of this variation may be the repertoire of presynaptic calcium channel subtypes, which can vary between synapses (Reid et al., 1997; Nimmervoll et al., 2013). In support of this, individual boutons displayed differential sensitivity to pharmacological blockade of Ca_V_2.1 and 2.2 channels in both calcium influx measured by GCaMP3 and exocytosis measured by sypHTomato, when these reporters were co-expressed in the same cell (Li and Tsien, 2012). Similarly, in another study in which calcium and vesicle dynamics were imaged in separate cells, pharmacological inhibition of Ca_V_2.1 or 2.2 led to variable blockade of neurotransmitter release between individual synapses, although this did not alter the relationship between calcium and exocytosis measured across the synapse population (Ariel et al., 2012). However, neither of these studies directly examined the relationship between channel subtypes and the calcium dependence of release in individual boutons, which could be addressed using sypHy-RGECO.

An alternative possibility is that individual boutons differ in the levels or components of the molecular machinery that links calcium influx to vesicle release. Of the markers that we examined using *post hoc* immunofluorescence, the level of the calcium sensor synaptotagmin-1 was not related to the ΔG/ΔR ratio, although a weak correlation was observed with the level of RIM, an active zone protein with roles in the localization of calcium channels and vesicle docking (Han et al., 2011). Whilst absolute RIM levels are only weakly predictive of the ratio, it is possible that more nuanced features such as the size and shape of the active zone relative to the bouton volume, and positioning of elements such as calcium channels within it may affect the calcium dependence of release at individual synapses. Combining functional imaging with structural imaging by immunofluorescence, super-resolution, or electron microscopy will enable investigation of these possibilities. Such methods could also be used to examine if differences in the calcium dependence of release exist in different cell types, or to study the relationship between presynaptic function and postsynaptic properties at individual connections.

Finally, it would be interesting to examine if the calcium dependence of release is fixed for an individual synapse or is subject to regulation. Calcium influx and vesicle exocytosis have been measured individually after chronic changes in network activity, and the changes observed indicate that the relationship between the two is not altered at the population level following homeostatic plasticity (Zhao C. et al., 2011). However, this is yet to be studied simultaneously in single synapses. Furthermore, it is unknown if short-term plasticity mechanisms involve changes in the efficiency with which calcium triggers vesicle fusion. Tools such as sypHy-RGECO will enable optical interrogation of these questions.

Several spectrally distinct genetically encoded reporters of calcium and vesicle recycling have been developed, offering the opportunity to combine probes with different characteristics. We selected to combine sypHy and R-GECO1 for several reasons; synaptophysin has previously been shown to tolerate addition of both pHluorin in the intravesicular loop with a second fluorophore at the C-terminus (Rose et al., 2013), and both sypHy and SyRGECO have previously been characterized individually (Granseth et al., 2006; Walker et al., 2013). SypHy has a superior signal-to-noise ratio than synaptopHluorin and whilst VGLUT-pHluorin offers further improvement in this aspect (Voglmaier et al., 2006), there is a risk that VGLUT1 overexpression may cause an increase in quantal content (Wojcik et al., 2004; Wilson et al., 2005), and therefore alter the strength of synaptic connections. Additionally, although VGLUT based probes have been used in inhibitory neurons, it may impart GABAergic boutons with unwanted properties resulting from the overexpression of a transporter not usually found in these neurons. R-GECO1 is a sensitive calcium indicator with a wide dynamic range that exhibits a larger fluorescence change to field stimuli than the RCaMP1 sensors (Akerboom et al., 2013; Walker et al., 2013). RCaMP2 (Inoue et al., 2015) offers greater sensitivity than R-GECO1 but saturates at approximately 8 APS and was therefore less suitable for combination with sypHy, for which longer stimulus trains are typically used. The ΔF/F responses of R-GECO1 are also linear over a greater range of calcium concentrations than RCaMP1 sensors (Akerboom et al., 2013). When combined in the single molecule sypHy-RGECO, both reporters displayed a linear increase in the amplitude of the response across the range of 1–20 AP stimuli delivered at 20 Hz, but at higher stimulus strengths the RGECO response became saturated. Whilst sypHy responses continued to increase to both 40 and 100 AP stimuli, the amplitude of these responses will be affected by the decrease in fluorescence caused by endocytosis and reacidifcation of vesicles. The dynamic ranges of the two reporters are therefore well matched and suited to reporting activity up to 20 action potentials.

One potential disadvantage of combining R-GECO1 with green sensors of vesicle exocytosis is its photoswitching behavior in response to 488 nm light (Akerboom et al., 2013). Indeed, we found that blue light exposure in our imaging conditions did cause an offset in the basal RGECO fluorescence, as well as a reduction in the calcium response. However, these decreases scaled linearly with the original R-GECO1 response, and both the kinetics of the calcium transient and the range of responses across synapses were preserved. SypHy-RGECO is therefore a useful addition to the expanding toolbox of genetically encoded reporters, and will enable a detailed study of the relationship between calcium influx and vesicle dynamics at individual synapses. Whilst we have shown proof of principle in hippocampal synapses *in vitro*, this reporter could also be used to study these processes in more intact systems, such as brain slices and *in vivo*.

## Author Contributions

JB and RJ designed experiments, RJ performed experiments, analyzed data, and drafted the manuscript, JB and RJ edited and approved the manuscript.

## Conflict of Interest Statement

The authors declare that the research was conducted in the absence of any commercial or financial relationships that could be construed as a potential conflict of interest.
