# Supplementary figures and images for: Visualizing Presynaptic Calcium Dynamics and Vesicle Fusion with a Single Genetically Encoded Reporter at Individual Synapses
Source: Front Synaptic Neurosci. 2016 Jul 26;8:21. doi: 10.3389/fnsyn.2016.00021 (PMC4960916; doi:10.3389/fnsyn.2016.00021)

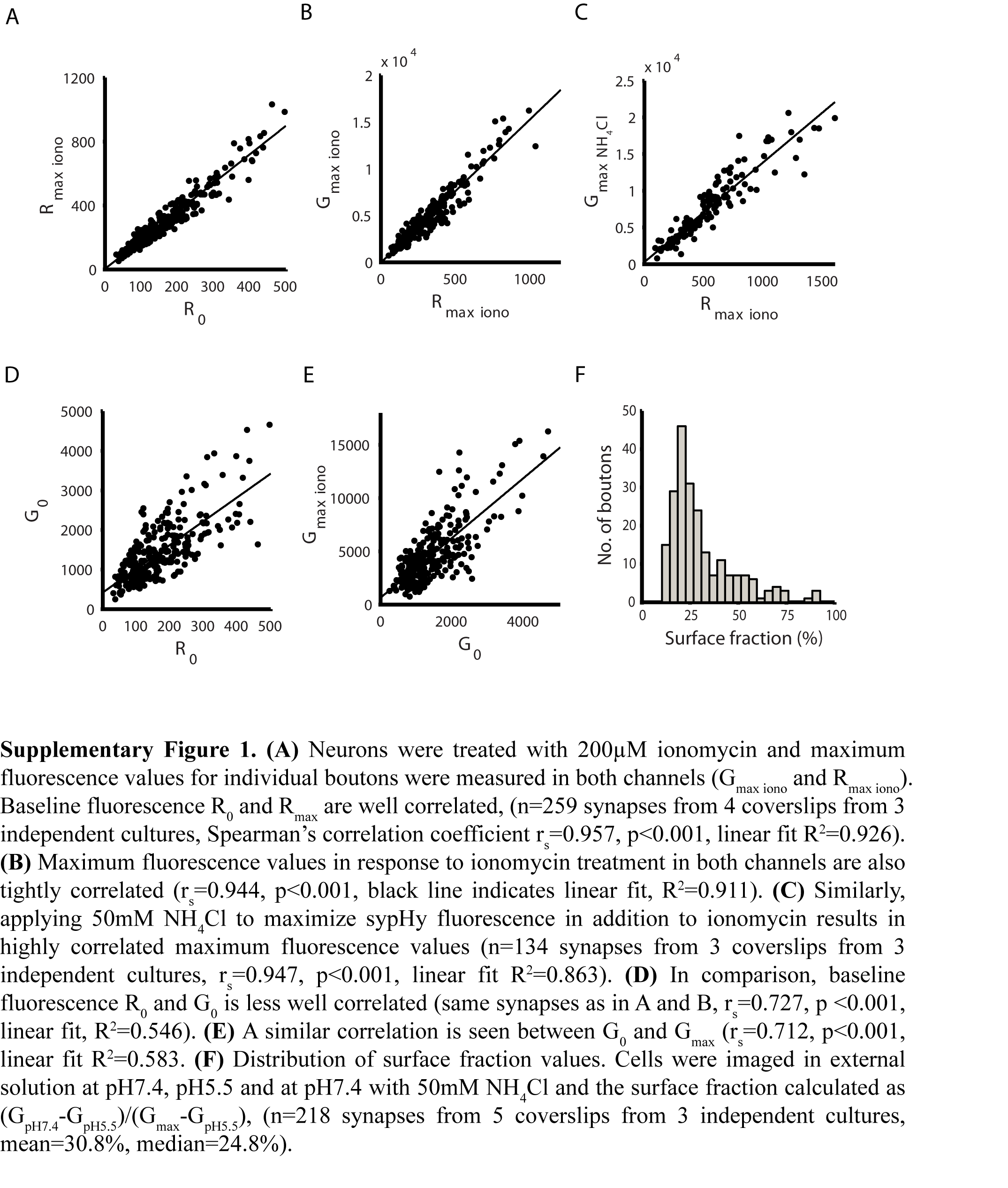

Supplement: Supplementary file 1 [file Image_1.TIF]

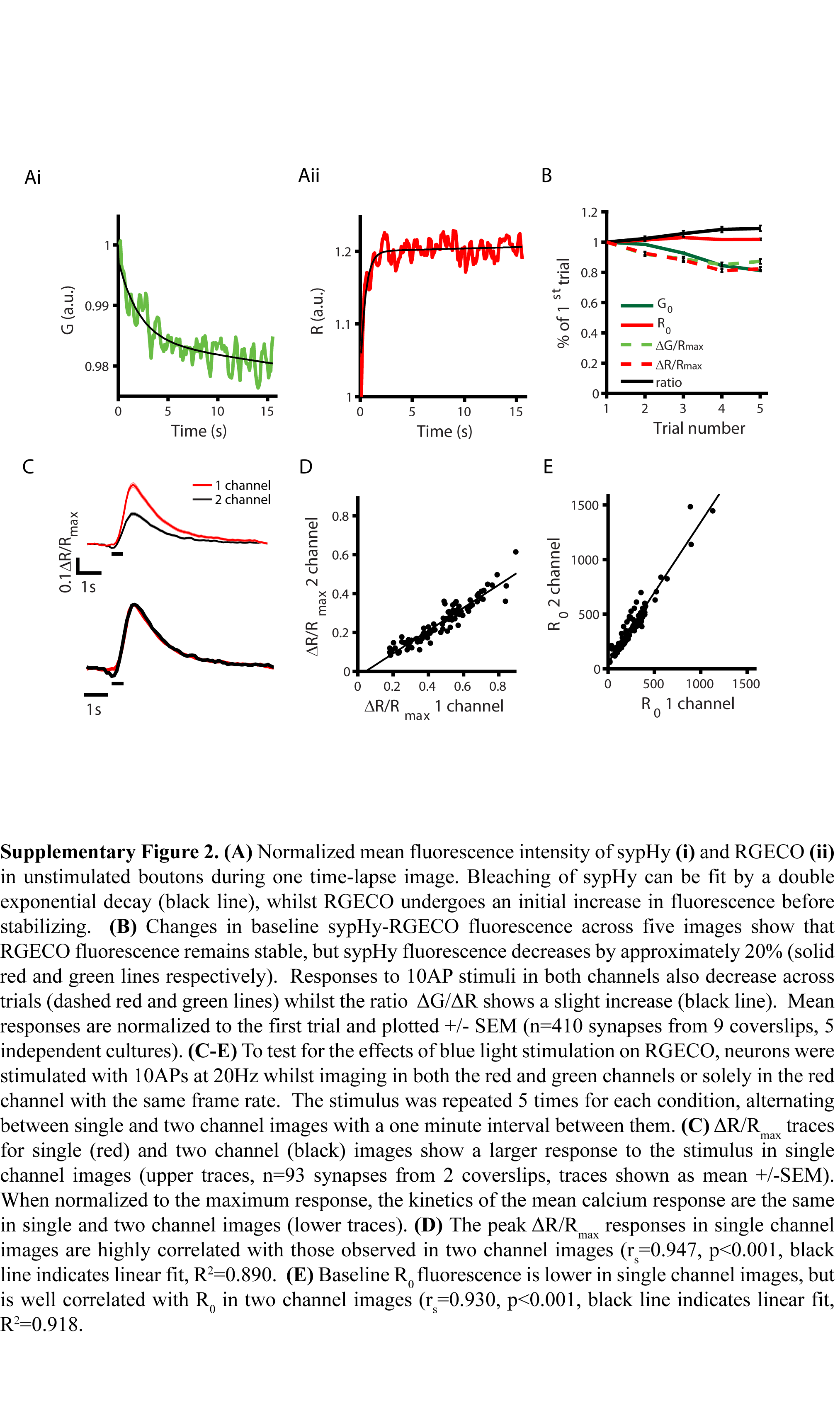

Supplement: Supplementary file 2 [file Image_2.TIF]

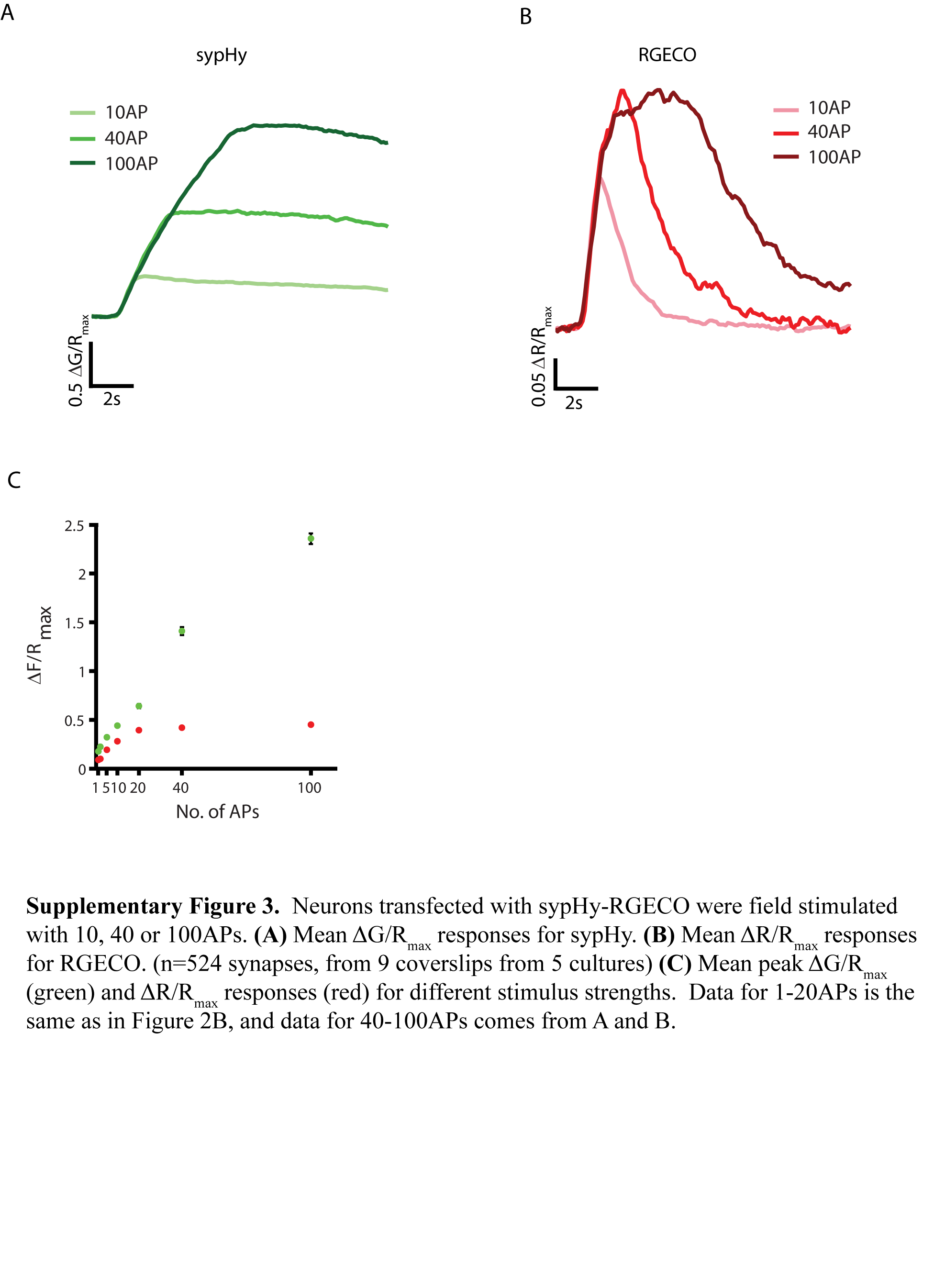

Supplement: Supplementary file 3 [file Image_3.TIF]

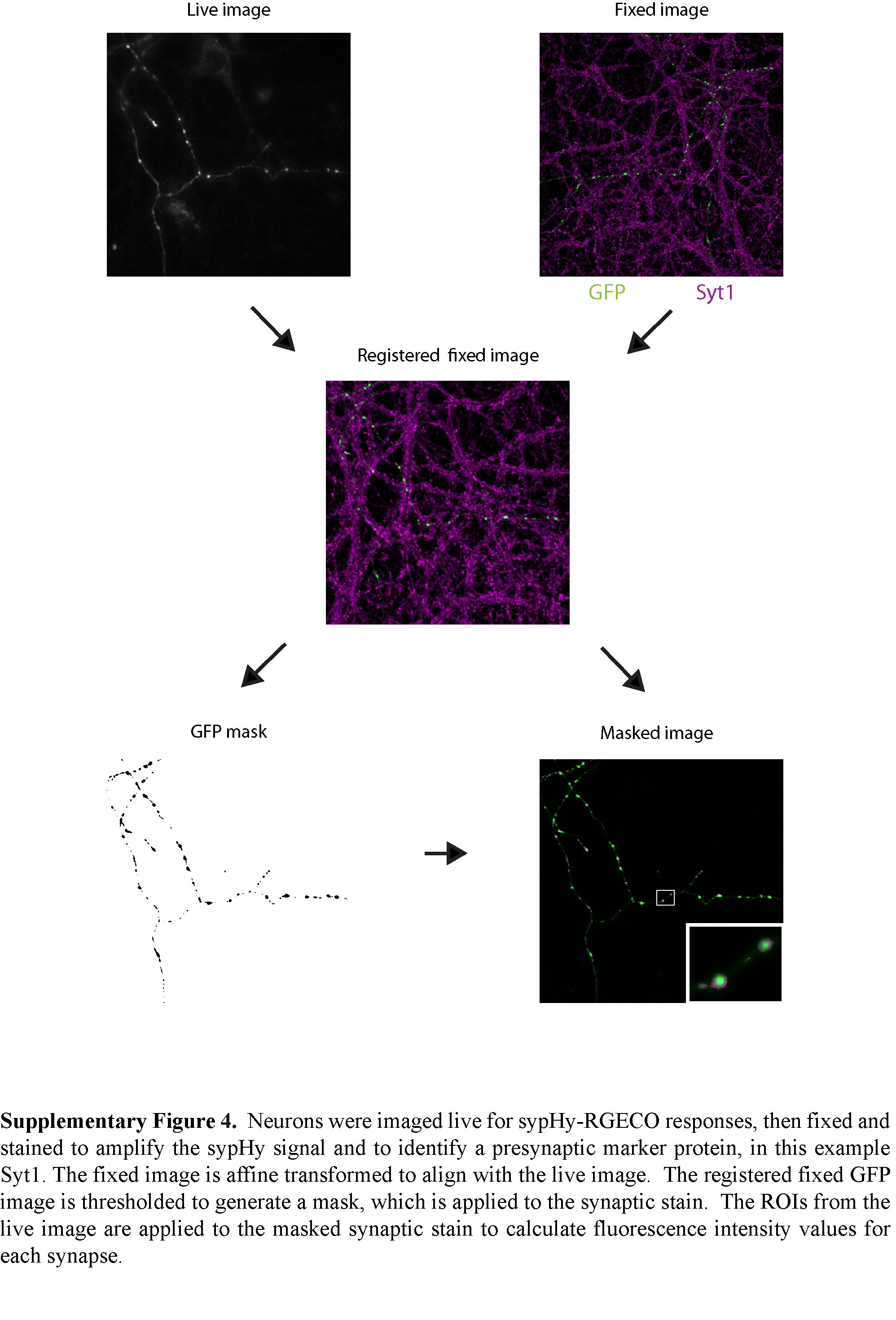

Supplement: Supplementary file 4 [file Image_4.TIF]
